# Deciphering waste bound nitrogen by employing psychrophillic *Aporrectodea caliginosa* and priming of coprolites by associated heterotrophic nitrifiers under high altitude Himalayas

**DOI:** 10.1038/s41598-022-12972-1

**Published:** 2022-06-10

**Authors:** Tahir Sheikh, Zahoor Baba, Ali Mohd Yatoo, Basharat Hamid, Sadaf Iqbal, Fehim Wani, Sabah Fatima, Saleh Alfarraj, Mohammad Javed Ansari

**Affiliations:** 1 Division of Agronomy, FoA, Sher-E-Kashmir University of Agricultural Sciences and Technology Kashmir-Wadura, Sopore, 193201 India; 2 Division of Basic Science, FoA, Sher-E-Kashmir University of Agricultural Sciences and Technology Kashmir-Wadura, Sopore, 193201 India; 3 Department of Environmental Sciences, University of Kashmir, Srinagar, 190001 India; 4 Faculty of Environment, University of Waterloo, Waterloo, Ontario Canada; 5 Division of Agricultural Economics & Statistics, FoA, Sher-E-Kashmir University of Agricultural Sciences and Technology Kashmir-Wadura, Sopore, 193201 India; 6 Zoology Department, College of Science, King Saud University, Riyadh, 11451 Saudi Arabia; 7 Department of Botany, Hindu College Moradabad (Mahatma Jyotiba Phule Rohilkhand University Bareilly), Moradabad, 244001 India

**Keywords:** Ecology, Microbiology, Environmental sciences

## Abstract

Himalayan ecosystem is characterized by its fragile climate with rich repositories of biodiversity. Waste collection and disposal are becoming increasingly difficult due to topographical variations. *Aporrectodea caligenosa*, a versatile psychrophillic soil dweller, is a useful biocatalyst with potent bio-augmented capability for waste treatment at low temperatures. Microcosm experiments were conducted to elucidate the comprehensive nature of biogenic nitrogen transformation to NH_4_^+^ and NO_3_^−^ produced by coupling of earthworm-microbes. Higher biogenic recovery of NH_4_^+^-N from coprolites of garden soil (47.73 ± 1.16%) and Himalayan goat manure (86.32 ± 0.92%) with an increment of 14.12 and 47.21% respectively over their respective control (without earthworms) with a linear decline beyond 4th week of incubation was reported. NO_3_^–^-N recovery progressively sustained in garden soil and goat manure coprolites during entire incubation with highest 81.81 ± 0.45 and 87.20 ± 1.08 µg-N g^−1^dry weight recorded in 6th and 5th week of incubation respectively and peak increments as 38.58 and 53.71% relative to respective control (without earthworms). Declined NH_4_^+^–N in coprolites at low temperature (15.0 ± 2.0 °C) evidenced increased nitrification rates by taking over the process by abundant nitrifying microbes. Steady de-nitrification with progressive incubation on an average was 16.95 ± 0.46 ng-N g^−1^ per week and 21.08 ± 0.87 ng-N g^−1^ per week compared to 14.03 ± 0.58 ng-N g^−1^ per week and 4.50 ± 0.31 ng-N g^−1^ per week in respective control treatments. Simultaneous heterotrophic nitrification and aerobic denitrification (SHNAD) was found to be a prominent bioprocess at low temperature that resulted in high and stable total nitrogen and nitrate accumulation from garden soil and goat manure with relative recovery efficiency of 11.12%, 14.97% and 14.20%; 19.34%. *A. caligenosa* shows promising prospects for mass applicability in biogenic N removal from manure of Himalayan goat.

## Introduction

Potential psychrophillic class of soil macrofauna are earthworms (Endogeic and Anecic species) that exploited deeper mineral soil beds, and thereby remodel and restructure nutrient pools in uppermost soil layers (hereinafter: geo-engineering earthworms)^1^. *Aporrectodea caliginosa* (Endogeic) is a non-permanent horizontal burrower and lives in the uppermost soil horizons^2,3^, ingesting enormous load of soil, rich in organic matter, leaving the casting as a key input into the soil and contributing biogeochemical cycles. Such earthworms have a direct impact on soil nutrient status and microbial population (GAPs i.e., gut associated processes) or an indirect impact (CAPs i.e. coprolites associated processes)^4,5^. They have the capacity to transform as well as restructure nitrogen (N) pools^6,7^ and geo-engineering by earthworms may have considerable effects on arctic soils^1^. Earthworms may influence the entire soil food web^8,9^ with significant influence on soil structure through horizontal and vertical drillosphere burrows and castings^10^ escalating the distribution and community composition of the temperate soil micro flora^11–13^. In temperate ecosystems earthworm cast production can range from 36 to 250 tons ha^−1^ year^−1^^14^. Earthworms represent biocatalysts of class oligochaeta that stimulate N mineralization^15–17^ and can bring about augmented nitrification^18,19^ or denitrification owing to their gut microbial community^20,21^. Earthworms stimulate microbial processes for recovery of N from organic compounds^22^. Moreover, microorganisms thrive in earthworm galleries because of transit through the worm gut and presence of lavish mucus and casts^23,24^. Biodegradable or perishable organic wastes could be converted into stable humus-like substances by way of vermicomposting^25^ as an end product which is a good organic fertilizer^26^ containing abundant humic acids and beneficial microbes^27^. Vermicoprolites have been observed to encompass elevated levels of NH_4_^+^, NO_3_^−^, Mg, P and K relative to surrounding soil^13,28,29^. The nitrogen transformation is an important component of waste conversion during composting and vermicomposting processes^30–32^ and processes like nitrification and denitrification are the key activities contributing to nitrogen cycle, playing an important role in nutrient supply, (NH_4_^+^-N), volatilization and greenhouse gas (GHG) emissions for various ecosystems^30,33^. Most of the reported heterotrophic nitrifying-aerobic bacteria are mesophilic bacteria, carrying out nitrification and denitrification at temperatures ranging from 15 to 37 °C^34,35^. Temperate microbes have obvious physiological advantages^36^ and unique ability to perform nitrification and denitrification synchronously^37^ and improve the overall nitrogen removing efficacy at lower temperatures. Guraz valley, a fragile cold ecosystem, located in high Himalayas with prominent features of hill agriculture and diverse habitats rich in biodiversity. The disparate topographic features offer microhabitat for a variety of organic herbal crop species to grow in the main cultivated area while woody *Betula pendula*, *Pinus roxburghii*, *Quercus robur* and *Cedrus deodara* grow in the surrounding forests^38^. All the three ethnic Himalayan communities (nomadic Bakerwals, seminomadic Gujjars and semi-sedentary Kashmiri shepherds) depend on natural resources^38^. Since, there is very insufficient and scanty information regarding wastes recycling and recovery of nitrogen nutrient in the Himalayan ecosystem. Nitrogen recovery from waste material has now become a pivotal issue globally (Wang et al. 2015; Chen et al. 2016). Open dumping of bio-wastes is considered a potent source of nitrogen pollution in different forms viz; NH_3_ and N_2_O to air and N-NO_3_^−^ to ground and surface water resources^39,40^. Changes in transformation/emission rates are affected by the soil temperature, because it affects the activity of urease, nitrifier communities, and nitrification rate in the soil. Transition in NH_4_^+^-N, NO_3_^−^-N and total nitrogen in the interim of vermicomposting had been reported, however, divergence and succession of N-transformation process through involving functional psychrophillic microbes and earthworms were seldom reported. Therefore, the information on cold tolerant earthworms and associated nitrifying and denitrifying microbial species can furnish novel insights into the N transformation during the vermicomposting process in cold habitats. Moreover, the positive priming through the endogeic geophagous earthworm’s influence is expected to foster the recycling of nutrients, especially organic carbon, nitrogen and phosphorus^41,42^. The present study, which is perhaps the first of its kind under cold habitat of Guraz valley in Kashmir region, will pledge cognition and contribute to sound understanding of mineral N dynamics using psychrophillic *A. caligenosa* indigenous to the Guraz valley, with the goal as (i) o determine the involvement of coprolite associated microorganisms in bioconversion of N, (ii) to enumerate the changes in N-NH_4_^+^, N-NO_3_^−^ and (iii) specifically, recycle and evaluate the impact of locally available garden soil and Himalayan goat manure on physico-chemical flux’s in coprolites resulting in minimizing nitrogen pollution in temperate ecosystems.

## Results and discussion

### Ammonium and nitrate dynamics

**Figure 1:**
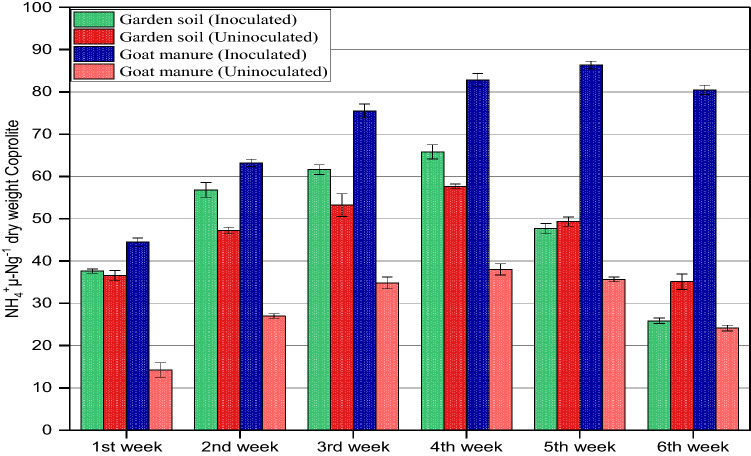
NH_4_^+^ content in coprolites with and without *A. caliginosa* from garden soil (GS) and goat manure (GM) at different intervals of the experiment.

**Figure 2:**
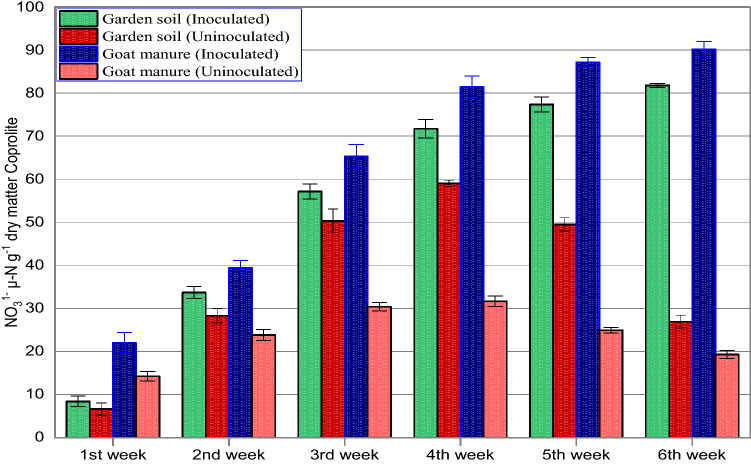
NO_3_^−^ content in coprolites with and without *A. caliginosa* from garden soil (GS) and goat manure (GM) at different intervals of the experiment.

The analysis performed on coprolites of *Aporrectodea caliginosa* evidenced that ammonium (NH_4_^+^-N) concentration after one week of incubation were significantly affected by the source of food (Fig. 1). As evident from Fig. 1 the concentration of NH_4_^+^-N in all the earthworm released coprolites increased steadily upto the 4th week of the vermicomposting process, however, coprolites of goat manure (GM) shows continuous increase in NH_4_^+^-N content upto the 5th week. The NH_4_^+^-N concentration in GM coprolites was significantly (*p* < 0.05) higher than GM_(control)_ concentration upto 5th week of incubation, after which the NH_4_^+^-N concentration began to fall steadily until the 6th week of incubation. Further, all treatments showed decline in the NH_4_^+^-N concentration after 4th week continued upto the 6th week of incubation. It was observed that after 6 weeks of vermicomposting the NH_4_^+^-N concentration in the coprolites was in the order; GM_(control)_ (24 µg NH_4_^+^-N g^-1^ DW) > GS_(Control)_ (35 µg NH_4_^+^-N g^–1^ DW) > GS (38 µgNH_4_^+^-Ng^−1^ DW) > GM (80 µg NH_4_^+^-N g^−1^ DW). Our results reflect that the gross transformation rate of N-NH_4_^+^ responded differently based on substrate type and feeding preference of *A. caliginosa*. GM aids gut associated microbes and was found to accelerate the mineralization of N to NH_4_^+^. The elevated NH_4_^+^-N levels in coprolites might be due to various nitrogen-containing compounds released by earthworm metabolism through muco proteins and urine. Previous studies also suggest that adult earthworms enhance ammonification^43,44^ by breakdown of large volumes of organic residues with the aid of gut associated bacteria thereby increases biogenic NH_4_^+^^31,45^. The steady increase of NH_4_^+^ concentration in GM and GS coprolites is related to the initial carbon and nitrogen content of the substrate. However, subsequent decrease with incubation time was reflected by the conversion of part of NH_4_^+^-N to NO_3_^–^-N, influenced by the dominance of nitrifying chemolithotrops at low temperatures (16.5–2 °C) (Fig. 1). Earlier research also suggests that ammonification is a temperature sensitive microbial mediated mineralization process^46–48^ and an increase in temperature upto 25 °C will significantly increase the transformation rate of N^49^. The trend for nitrate (NO_3_^−^N) concentration varied between 8.37 and 81.88 µg NO_3_^–^N g^–1^DW in coprolites influenced by different treatments. The NO_3_^–^-N concentration of coprolites from earthworms fed on GM and GS was found higher. NO_3_^–^-N concentration showed a linear increase from the 1st to 6th week of vermicomposting from coprolites of both GM and GS with the highest values recorded as 90.2 µg NO_3_^–^N g^–1^ DW and 81.81 µg NO_3_^–^N g^–1^ DW respectively. The usefulness of earthworm^’^s gut microbes inflates the process of nitrification compared to the situation without earthworms^43,50^. The NO_3_^–^-N removal from the GM was exceptionally high and ranged between 22.03 to 90.20 µg- NO_3_ g^–1^DW with an average value of 64.24 µg- NO_3_ g^–1^ DW during the entire incubation period (Fig. 2). Similarly, for GS NO_3_^–^-N recovery ranges from 8.37 to 81.81 µgNO_3_ g^−1^ DW*.* Further, our findings reveal a linear increase in NO_3_^–^-N concentration from GM coprolites along the duration of incubation, which is assumed to be correlated with a higher concentration of NH_4_^+^-N in coprolites, which is a primary source for the nitrification process. Our results also suggest that continuous escalation in NO_3_^–^-N could be due to the involvement of heterotrophic bacteria and fungi in coprolites (Table 1) preferring a moderate temperature between 15 and 25 °C which also shows that heterotrophic nitrification process exceeds autotrophic nitrification. Previous research findings suggest that earthworm gut and castings tend to have more active heterotrophic microbes that positively influence the amounts of extractable NO_3_^–^-N in upper layer of soil^51–53^ moreover, increased NO_3_^−^ concentration in coprolites is a good indicator of the nitrification process and abundance of nitrifiers^23,25,54–56^. It is also reported that the heterotrophic nitrification has an optimum temperature requirement of 15 °C^57^ while, it exceeds over autotrophic nitrification if the temperature is increased to 35 °C^49^. Further, we confirm that the heterotrophic nitrification is an ecosystem dependent process which is directly dependent on substrate type, microbial diversity and abundance. The samples of study material yielded a large number of indigenous heterotrophic nitrifiers, which were determined to have high nitrogen removal abilities. Temperature stimuli, NH_4_^+^-N and NO3^–^- N content in substrates influenced the overall performance of heterotrophic nitrification process. Our previous research in the cold arid Ladakh and Kashmir valley backs up this argument. The influence of low temperature on heterotrophic nitrification at the experimental site (Guraz valley) is consistent with the previous findings, which demonstrated that an optimal temperature, substrate type and ecology conditions favor heterotrophic nitrification process^13,58,59^. Low concentration of NO_3_^−^N is related to low microbial dominance (nitrifying bacteria) in coprolites from control treatments of both the substrates suggesting the loss of potency in coprolites which inhibited the nitrification process. Lower functional redundancy in the nitrification process is due to the lack of earthworms in the control treatments which could not either enhance biogeochemical stability of organic matter or stimulate microbial activity (Table 1). Thus, the results clearly elucidated an amicable relationship between earthworm and heterotrophs which favors the heterotrophic nitrification. Previous studies support the increased mineralization of nutrients in earthworm coprolites relative to surrounding garden soil (without earthworm), which is associated with enrichment in liable compounds due to various factors such as, increased activity of nitrifying microbes^60^, digestion of earthworm could influence gut microbiome^61^ and earthworm-microbe association produces enzymes that are reported to increase NO_3_^−^ N and NH_4_^+^-N content in coprolites by 31 and 14% respectively^62–65^.

**Table 1:** Distributions of microbial isolates in the different cast-types.

Microbial isolates	Garden soil (control)	Goat Manure (control)	Goat Manure (inoculated with earthworms)	Garden soil (inoculated with earthworms)
Bacteria				
*Nitrosomonas sp*	+	−	+	+
*Bacillus sp*	+	+	−	+
*Citrobacter sp*	−	−	+	+
*Nitrosopira sp*	+	−	+	+
*Klebsiella sp*	+	−	−	−
*Pseudomonas sp*	+	−	+	−
*Proteus sp*	−	+	+	+
*Serratia sp*	−	−	+	−
*Staphylococcus sp*	−	−	+	+
Fungi				
*Aspergillus sp*	+	−	−	−
*Fusarium sp*	+	−	−	+
*Penicillium sp*	−	−	+	−
*Saccharomyces sp*	−	+	+	+

### Denitrification/ N_2_O emission during incubation

**Figure 3:**
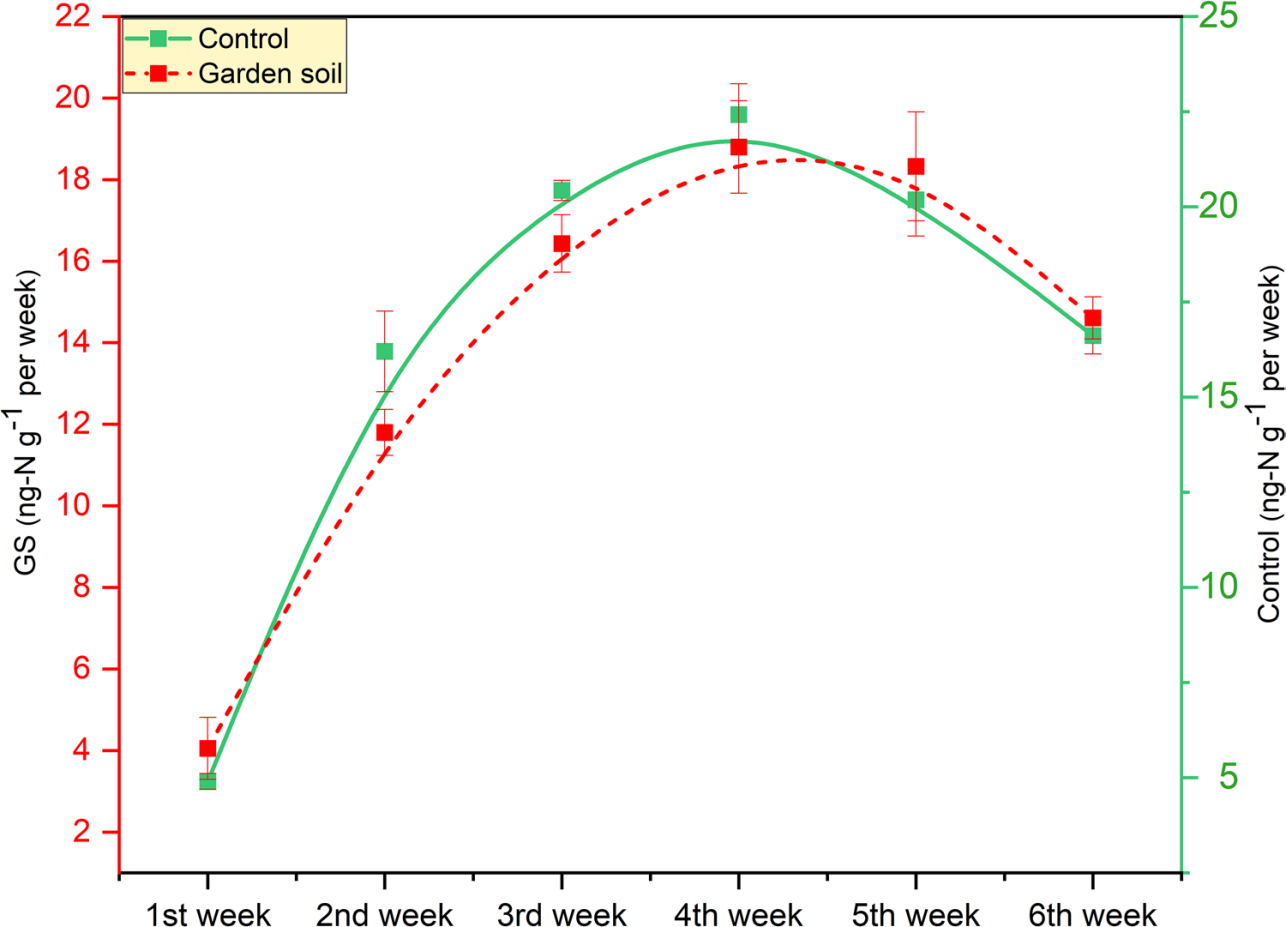
Temporal variation in denitrification rates (ng-Ng^−1^ per week) from coprolites of *A. caligonosa* by food source (garden soil).

The interaction between earthworms and denitrifers directly affect the nitrogen dynamics via nitrous oxide (N_2_O) fluxes. Our study reveals that denitrification predominantly turned out to be limited by low temperature and C supply. N_2_O emission rates from mesocosm surface coprolites (soil fed earthworms) ranged from 5.90 ± 0.20 ng-N g^−1^ (1st week) to 16.6 ± 0.48 ng-N g^−1^ (6th week) with an average of 16.95 ng-N g^−1^ (Fig. 3). However, the emission rates of N_2_O from the control treatments, on the hand were relatively low ranging from 4.05 ± 0.29 ng-N g^−1^ (1st week) to 14.60 ± 0.22 n g-N g^−1^ (6th week) with an average of 14.0 ng-N g^−1^ per week. N_2_O emissions showed a steady increase along with the incubation period except from the 6th week onwards, there was a downward trend, with an average increase of 17.03% in emission from coprolites of GS compared to GS_(control)_. Earlier research has shown that *A. caliginous* is capable to emit significant amounts of N_2_O emissions from the soil through different activities^66–68^. Earthworms have the potential to dramatically regulate the physico-chemical properties of their habitats and thereupon affecting the production of GHG^20^ however, denitrification is affected by a variety of environmental factors including availability of dissolved oxygen (DO), carbon (C), pH, temperature, denitrifying bacterial population and congregations of NO_3_^−^, NO^2−^ and S^2−^^31,69,70^.

**Figure 4:**
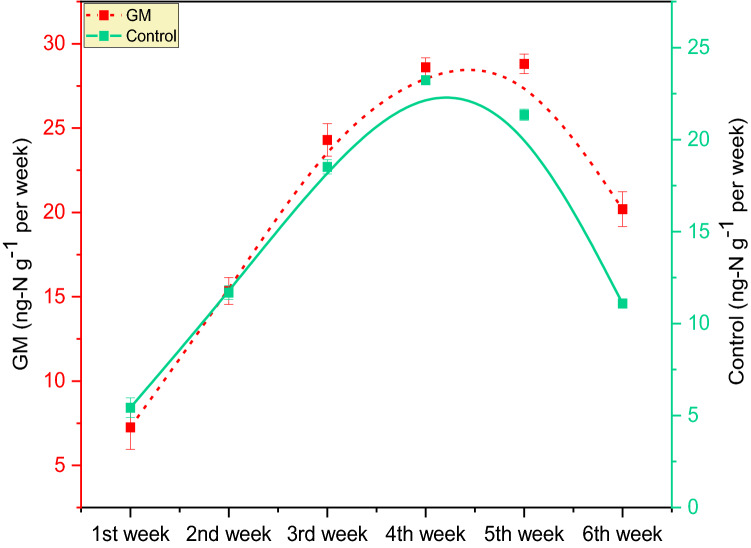
Temporal variation in denitrification rates (ng-Ng^−1^ per week) in casts from goat manure (GM) with and without *A. caliginosa*.

**Figure 5:**
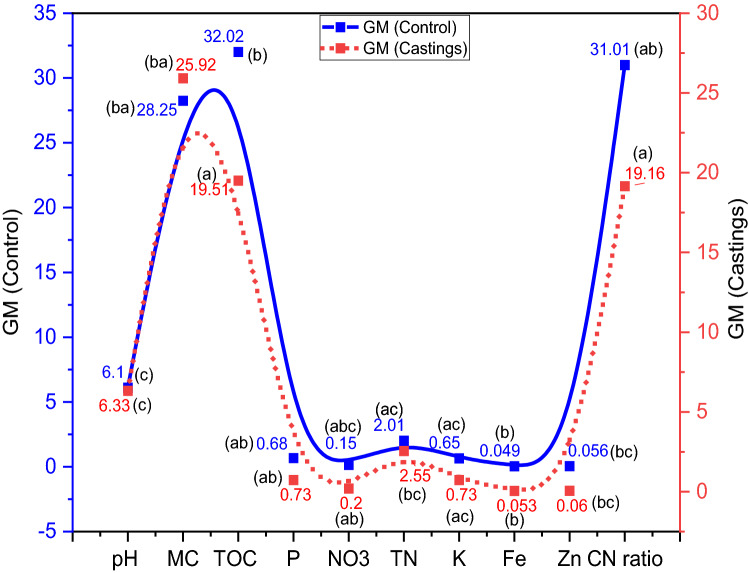
Physico-chemical characteristics of manure (initial) and coprolites of Goat manure (GM) with and without *A. caliginosa* (mean ± SD). Corresponding means followed with same letters are not significantly different at *P* < 0.05.

Under mesocosm condition, incubation of earthworm with GM stimulates the denitrification process, which showed a favorable relationship with N_2_O emission from manure coprolites ranged from 7.26 ± 1.32 ng-N g^−1^ (1st week) to 20.19 ± 1.03 ng-N g^−1^ (6th week) (Fig. 4). On an average N_2_O emission from coprolites of GM was 78.65% higher compared to GM_(control)_, suggesting that *A. caliginos*a is playing a critical role in conversion of excess NO_3_^−^ to N_2_O. It might be attributed to the higher initial availability of carbon in goat manure (Fig. 5), which constituted 70% of the total mass in this substrate, led to faster degradation and enhanced mineralization of N to NO_3_^−^. Limited reports are available about the mechanism involved in the combined relationship between the nitrifying and denitrifying microbes that help towards better understanding of the N-cycle in terrestrial ecosystem^71,72^. Bioconversion of cow dung, duck manure, kitchen waste by earthworms is reported to induce N_2_O emissions^54,73^; however, emission rates are significantly lower than thermophiles composting^74,75^. Vermicomposting under high moisture conditions were reported to decrease N_2_O and CH_4_ emissions by 25–36 and 22–26% compared to thermal composting^74^. Increased N_2_O emission in GM coprolite must be owing to the activities of denitrifying bacteria, as evidenced by significant (*p* < 0.05) difference in N_2_O emission between samples from GM and GM_(control)_ treatments. The research also found a link between nitrification and denitrification (Figs. 4, 8), and demonstrate how a heterogeneous microbial population and function may coexist. Previous research also found that potential denitrification rates were positively correlated with coexistence of aerobic and anaerobic microbes (denitrifers)^76–80^. In our study, low N_2_O accumulation was found in headspace of bottles with no C_2_H_2_ or with low concentration for both control treatments of GS_(control)_ and GM_(control)_. However, significant (*p* < 0.05) difference in N_2_O emission was evidenced between the two control treatments GS_(control)_ and GM_(control)._ On an average, N_2_O produced from the coprolites fed on GM were 19.57% higher compared to castings obtained from GS.

### Physico-chemical analysis of worm casts

**Figure 6:**
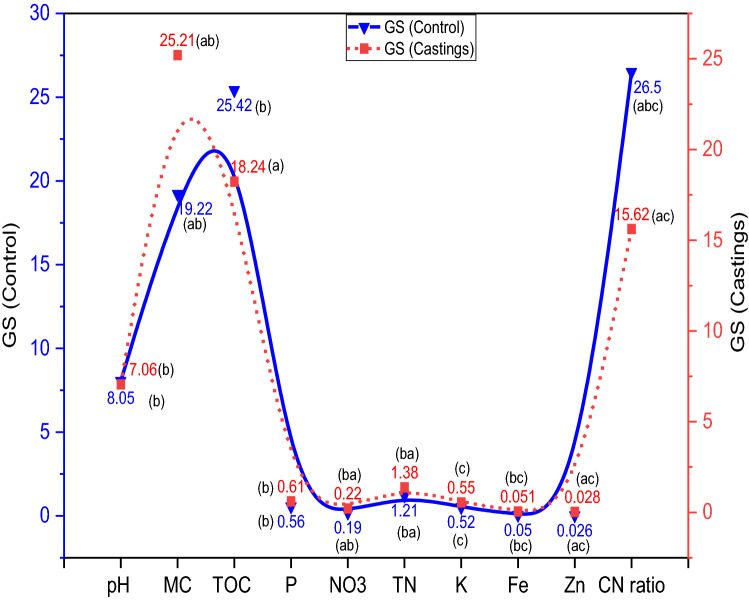
Physico-chemical characteristics of garden soil (initial) and coprolites of garden soil (GS) with and without *A. caliginosa* (mean ± SD). Corresponding means followed with same letters are not significantly different at *P* < 0.05.

**Figure 7:**
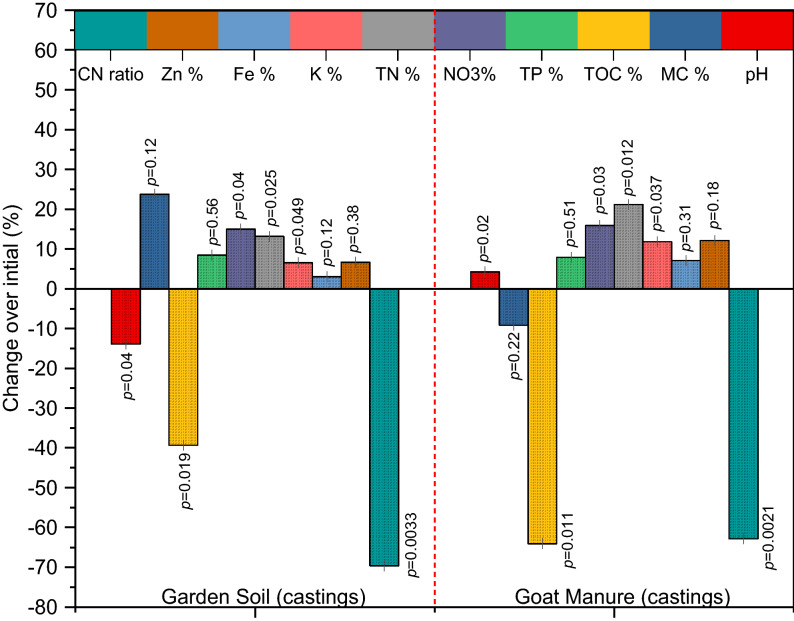
Percent change over initial in the physico-chemical characteristics of vermicompost.

Coprolites produced from GS and GM were rich in nutrients and *A. caliginous* had a substantial impact on the analyzed parameters. Significantly (*p* < 0.05) higher pH was observed in coprolites from GS and GM treatments relative to the GS_(control)_ and GM_(control)_ (Fig. 5, 6). The final pH values of both the coprolites from GS and GM were slightly acidic to alkaline attributed to gut microbial activity, indicating that these coprolites could be useful to remediate soil reaction. Vermicomposting of fruit and vegetable wastes^81,82^; seaweeds, sugarcane trash, coir pith amended with cow dung^83^; and flowering plants (*Lantana camara*)^84^ was also reported to produce vermicompost with a pH close to neutral. Analyses of variance for total organic carbon indicated significant (*p* < 0.05) differences between the coprolites from GS (18.24%) and GM (27.18%) with their controls GS _(control)_ (29.42%) and GM_(control)_ (32.02%) respectively. A linear decline in total organic carbon from GS and GM coprolites is attributable to utilization of carbon by microbes during the entire process (Fig. 7). Previous reports have also mentioned the loss of 19–67% of carbon during the process of vermicomposting^85,86^ where dehydrogenase activity plays a key role in the hydrolysis of cellobiose and other disaccharides during vermicomposting process^82^. It has been also reported that the chief mechanism for the carbon loss from the substrates could be attributed to the respiration of earthworms and microorganisms during the decomposition and transformation of substrates^87,88^. The Fig. 7 depicts a significant (*p* < 0.05) increase in NO_3_^−^ by 15 and 39% in coprolites of GS and GM respectively, when compared to GS_(control)_ and GM_(control)_. Increased NO_3_^-^ content in coprolite is attributed to the significant influence of gut associated nitrifying microbes in the production of NH_4_^+^ which is a primary substrate for NO_3_^−^ yield, in addition, earthworm mucus and nitrogen-rich excretory secretions also contributed to NO_3_^−^ content. The NH_4_^+^ concentration is also correlated with the initial N content of waste substrates, which was 1.85 ± 0.04 and 2.14 ± 0.05% in GS_(control)_ and GM_(control)_, respectively (Fig. 7). Previous research have noted that earthworms may ameliorate the castings as a consequence of N transformation from wastes by associated microbes through bio-waste mineralization and gut N-fixation^21,89–91^. Total N concentration in the coprolites significantly (*p* < 0.05) increased and could be interpreted due to the factors such as: initial N content of the substrate; bioconversion efficiency; possible death of a few baby worms; secretions of mucus, addition of nitrogenous substances during the entire process. Gut and skin of earthworm can secrete nitrogenous compounds which is also one of the reasons for enriched N content in the end product of the process^86,92^. At the end of vermicomposting process, the total phosphorus (TP) recovery was found 6.15% in GM and 8.19% GS from the respective substrates (Fig. 7). Increased P concentration in the GM castings could be due to secretion of various organic acids by related microbes and decomposition of substrates by earthworm, as corroborated with previous studies^93,94^. Table 1 shows that the bacterial population in both coprolites from GS and GM increased that includes *Aerobacter* sp., *Bacillus* sp., *Citrobacter* sp., *Escherichia* sp., *Klebsiella* sp., *Pseudomonas* sp., *Proteus* sp., *Serratia* sp. *and* *Staphylococcus* sp*.* The coprolites of GS and GM demonstrated significantly (*p* < 0.05) higher bacterial and fungal density compared to respective controls GS_(control)_ and GM _(control)_.

**Figure 8:**
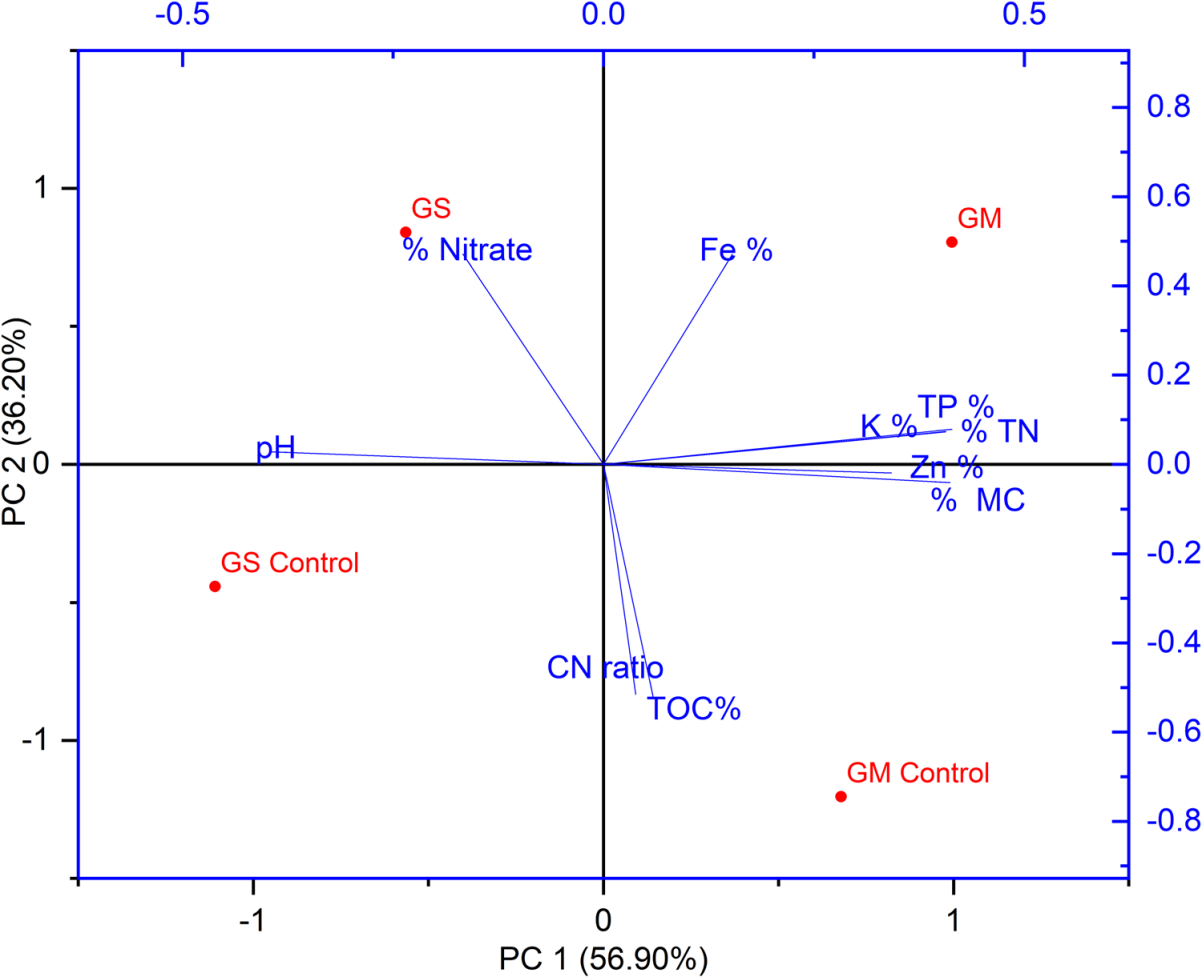
Principal component analysis of physico-chemical parameters in GS and GM coprolites compared to controls. The uninoculated treatments with *A. caliginosa* are referred to as the control.

The earthworm amended treatments demonstrated significantly (*p* < 0.05) higher bacterial density than GM_(control)_. Similarly, higher fungal density was also observed in earthworm inoculated coprolites of GS and GM. The fungal species included *Aspergillus* sp*.*, *Fusarium* sp*.*, *Penicillium* sp*.* *and Saccharomyces* sp. The presence of more bacteria and fungi in earthworm amended coprolites indicate that earthworms could favor and compliment the microbial communities during conversion of substrates. This is in accordance with earlier results of using mill waste^95,96^, forest litter waste^97^, sewage sludge and rice straw^31^ as substrates in vermicomposting which favors the microbial population. It has also been reported that presence of the earthworms in vermicomposting enhances the beneficial microflora and suppresses harmful pathogenic microbes^98^, that later enhance plant growth via the production of plant growth promoting compounds. Thus, the higher population of microbes in the earthworm castings could be due to the abundance of beneficial microbes in the earthworm gut. This concept was also supported by the findings of previous research^99,100^. The potential availability of nutrients in coprolites was revealed by regression analysis between the indices of physico-chemical properties of GM (Table 2). The Fig. 8 shows that the selected parameters were appropriate to determine the stabilization of coprolites in the current investigation. With a cumulative variance 93.10% and high rate of loading from first and second principal component, parameters MC, TN, TP, K, and Zn are strongly correlated with Fe, CN ratio, TOC in the first main component which reflects mineralization and breakdown potential of GM (goat manure). Nitrates and pH are negatively correlated with the second component. It can be inferred that *A. caligenosa* may have good impact on stabilization of coprolites with positive correlation to initial nutrient status of GM. It implies that coprolites produced from GM are superior to GS as a soil fertilizer. The loading of the variables on the components are presented in Table 3. Which indicates score data of extracted eigenvectors with a corresponding loading of 56.90 and 36.20% for principal component-1 and principal component-2 these loadings in have been computed from the correlation coefficients between these variables.

**Table 2:** Regression between indices of physico-chemical characteristics of goat manure (GM).

Parameter	Equation	R^2^
pH	$Y = - 0.0081X^{2} - 0.6860X + 2.56$	0.64
Moisture content	$Y = - 0.0487X^{2} - 3.4389X + 34.11$	0.43
Total organic carbon	$Y = 0.0457X^{2} - 1.9482X + 42.28$	0.85
Total phosphate	$Y = - 5.6127X^{2} - 6.9485X + 1.44$	0.62
Nitrate	$Y = - 0.6396X^{2} - 0.3569X + 0.122$	0.91
Total nitrogen	$Y = 3.778X^{2} - 14.975X + 16.90$	0.89

**Table 3:** Score data of extracted eigenvectors of principal component-1 (56.90% loading) and principal component-2 (36.20% loading).

Parameters	Coefficients of PC-1	Coefficients of PC-2
pH	− 0.39427	0.02769
% MC	0.34199	− 0.01981
TOC%	0.05875	− 0.52019
TP %	0.41406	0.07825
% Nitrate	− 0.16718	0.47157
% TN	0.40588	0.07231
K %	0.40687	0.07334
Fe %	0.1531	0.47115
Zn %	0.41179	− 0.04123
CN ratio	0.03815	− 0.51521

### Nitrification potential

**Figure 9:**
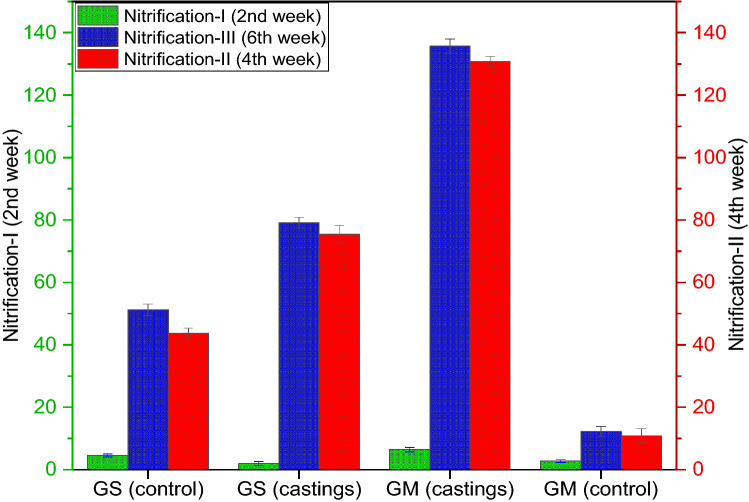
Effect of different substrates (garden soil and goat manure) on nitrification potential (three successive nitrification stages are designated as nitrification-I, nitrification-II, and nitrification-III) of coprolites at variable dates of incubation with and without *A. caliginosa*.

Replicated sampling analysis at intervals revealed an increase in potential nitrification rates, demonstrating a continuous nitrification process in coprolites in all the treatments except control (Fig. 9). The nitrification sampling in triplicates was done at three different stages of vermicomposting; nitrification-I (2nd week), nitrification-II (4th week) and nitrification-III (6th week) to estimate NO_3_^−^ levels. Nitrification rates increased over ammonification, as performed by the involvement of both nitrifying bacteria and fungi (Table 1). The mean potential nitrification rates of GS and GM coprolites at three stages of the nitrification process were 4.63, 43.46, 51.21 µN g^−1^DW and 6.48, 130.43 135.71 µN g^−1^DW respectively that were significantly (*p* < 0.05) different from the potential nitrification rates of the respective GS_(control)_ & GM_(control)_ (Fig. 9). Abundances of nitrifying bacteria and nitrifying fungi increased nitrification during the entire incubation period, apart from that nitrification potential by earthworm, it also depends on the initial N content of organic substances used by earthworms as a source of food. The study evidenced that unlike other microbial processes, nitrification progressed with a steady increase in NO_3_^−^ concentration at all the three stages of nitrification process. In contrast, the net rate of nitrification was comparatively low in the GS_(control)_ & GM_(control)_. Previous studies indicate that earthworm castings have strong associated activity for nitrification^101–103^. Our findings, support the hypothesis that earthworm activities with consumption of N-rich food material could increase the nitrification process in coprolites which is also supported by earlier research^44,91,104^.

## Materials and methods

### Site description and cold tolerant earthworms

**Figure 10:**
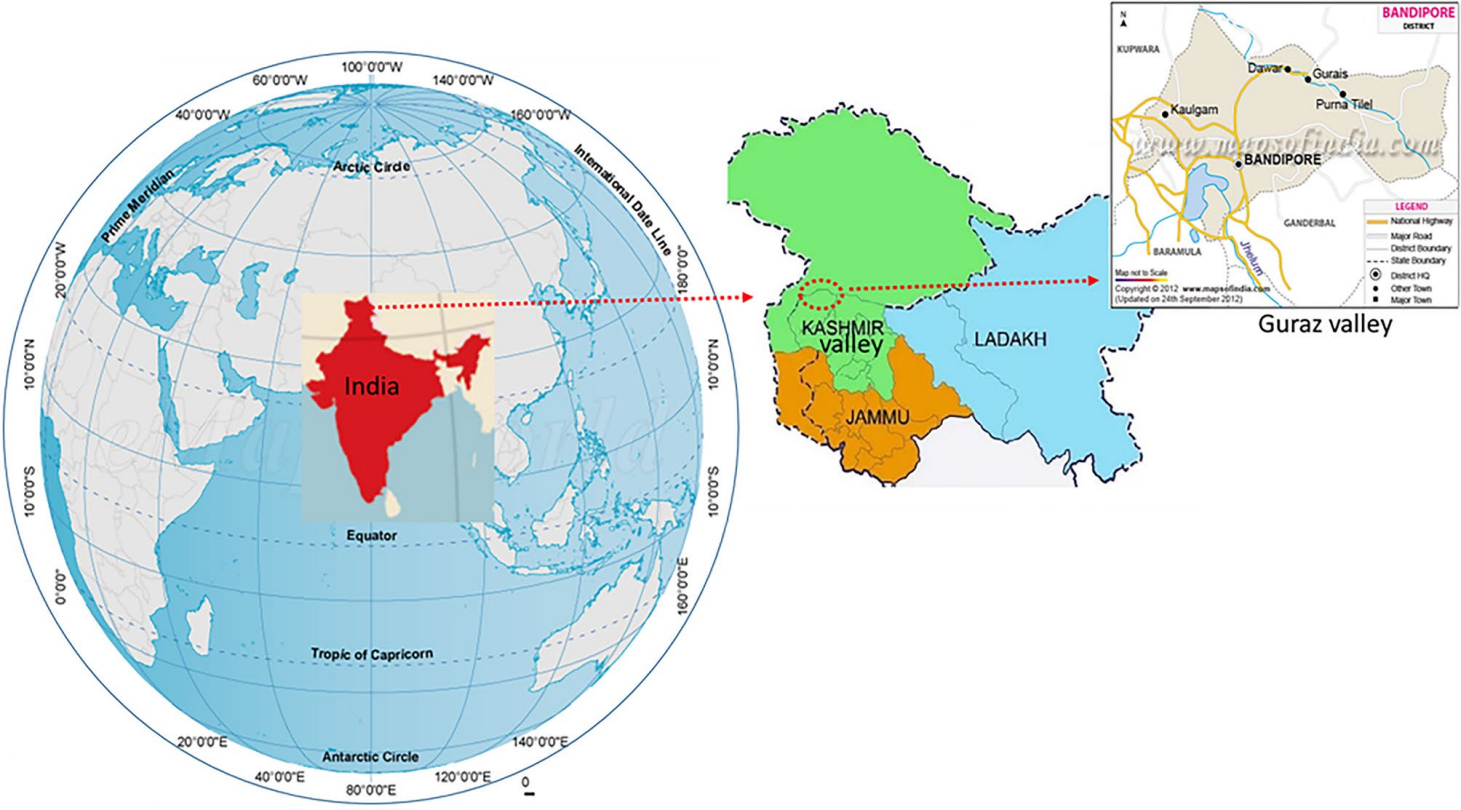
Study area map of Guraz valley in Kashmir region-India (Microsoft power point 2013; https://worldmapblank.org/wordpress/wp-content/uploads/2021/08/Location-of-India-on-the-World-map.pdf).

Geophagous endogeic earthworms *Aporrectodea caliginosa* (*A. caliginosa*) were obtained from the Mountain Agricultural Research and Extension Station—Izmarg, Guraz valley (74.73° E, 34.64° N; altitude 2580 masl) of District Bandipora, Jammu and Kashmir-India (Fig. 10). The annual mean temperature and precipitation at experimental site are 15.6 °C and 2200 mm, respectively.

### Experiment

**Figure 11:**
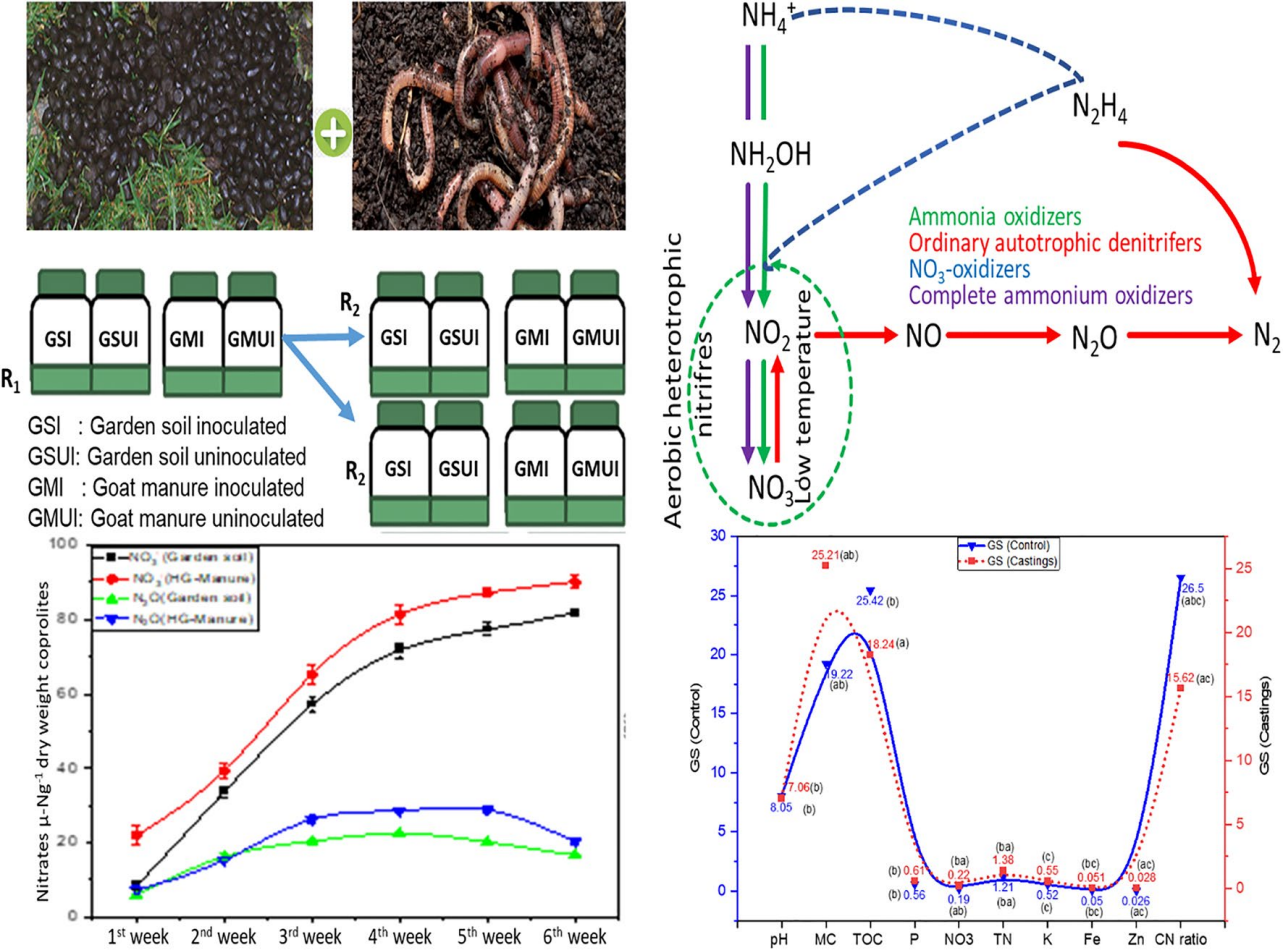
Graphical abstract: Microcosm design depicting set up for recovery of biogenic nitrogen with simultaneous heterotrophic nitrification and aerobic denitrification (SHNAD) and determination of nitrification potential of garden soil and Himalayan goat manure employing *Aporrectodea caliginosa*.

The present study used *A. caliginosa*, which is found in the garden and coniferous forest soils of Guraz valley^105^. Garden soil (GS) from a field planted with maize + beans and goat manure (GM) from Bakerwal (Himalayan shepherds with rearing Himalayan goats) were used as substrate treatments in triplicates with corresponding control as designated in Table 4. Earthworms were placed in 12-mesocosms (polyvinylchloride–PVC) with a diameter of 550 cm^3^ filled with substrates separately (Fig. 11). The activity of nitrogen dynamics from the earthworm coprolites was sampled three times in a week. Mesocosms were filled with the same feeding material and inoculated with ten (10) non-clitellated young worms for 42–45 days. Surface coprolites samples were shifted to petri dishes with moistened filter paper for further analysis. The mesocosms were placed under ambient light, with an average air temperature and relative humidity of 16.8 ± 1.5 °C and 67 ± 4% respectively, to ensure similar microclimatic conditions during the course of the experiment.

**Table 4:** Treatment combinations of garden soil and goat manure.

S. no	Labels	Substrate treatments
1	GS	Garden soil (inoculated with *Aporrectodea caliginosa*)
2	GM	Himalayan Goat manure (inoculated with *Aporrectodea caliginosa*)
3	GS_(control)_	Garden soil (Un inoculated)
4	GM_(control)_	Himalayan Goat manure (Un inoculated)

### Measurements

Surface coprolites of *A. caliginosa* were collected three times a week from each mesocosm, oven dried at 40 °C for 36 h and weighed for further examination. In the first week, a few dead earthworms were found on substrate surface and promptly replaced with new living specimens. Coprolites were analyzed for ammonium (NH_4_^+^) and nitrate (NO_3_^−^) concentration by extracting with 2 M KCl with KCl: coprolites ratio of 4:1. The mixture obtained was mechanically shaken for 3 h at 24 °C followed by filtration. NH_4_^+^ was determined by the indophenol blue method (IBM) (Keeney and Nelson 1982) using an automated unit (Sysmex BX4000) while NO_3_^−^ by the cadmium reduction method (CRM)^106^. Triplicate surface coprolites (3 cm diameter) from each mesocosm were collected and evaluated for NH_4_^+^and NO_3_^−^ at each sampling time, and the results were reported on dry weight basis. At three distinct stages of experiment, the nitrification potential of coprolites from both types of substrates was determined using the method^107^ as: Coprolites samples (2–4 g fresh weight) from both substrates and control were put into flasks with 25 ml (NH_4_^+^ & SO_4_^–2^) solution. Foam plugged flasks were shaken mechanically at 25 °C for 2 h. A mixture sample of 10 ml was taken for N_2_O analysis. Flasks were then exposed to aerobic conditions for two days followed by filtration and the amount of N_2_O was measured. The difference between the initial and final readings of N_2_O was recorded as the nitrification potential. Nitrification rates were expressed on a dry weight basis. During the experiment, denitrification rates were determined three time (2nd, 4th and 6th weeks) using surface coprolites taken from each mesocosm. All castings were removed from mesocosm surface two days before coprolite sampling, so all coprolite samples evaluated for denitrification in the experiment were ≤ 2 days old. The acetylene block method^108^ was used to determine denitrification rate as: Coprolite sample (2 g fresh weight) of *A. caliginosa* from each mesocosm surface were placed in10 ml test tubes fixed with headspace, and 1.0 ml of hydrogen and carbon was added to the headspace of each test tube to inhibit the reduction of N_2_O to N. N_2_O production was measured every 3 h for 14 h at 16 ± 2.5 °C. Modified automated method^109^, was used to determine the N_2_O.

### Coprolite microbiome

For differential analysis of the micro flora, coprolites samples were sub-sampled. In addition, earthworms were put under starvation for two days in petri dishes with 1% sterile agar. This much time was sufficient to get the transit microbes come over the agar. Coprolite samples were dissolved into 10 ml of sterile 0.85% NaCl and stirred vigorously for 20 min using the method^110^ described as: Suspension was diluted by the serial dilution method using a dilution factor of 10^–1^–10^–10^ to make out the development of microbes on agar nutrient plates. Enumeration of microorganism was carried out by pour plate method on nutrient agar. Microbial samples were incubated for 24 h at 20 ± 2 °C. Using the purified streak-plate technique, each isolate with similar morphological features was eventually relocated to a new nutrient agar plate until a single colony was established. Pure microbial colonies were characterized and identified by perceiving morphological features and bacterial cell shapes through the gram staining technique^111^. Nitrogen transformation activity of pure isolates were tested by using Kjeldahl method^112^, on the basis of generation of initial turbidity in flasks with nitrogen free medium.

### Chemical analysis

Coprolite samples were ignited in Muffle furnace at 500 °C for 90 min for determination of organic carbon using the method as described by Nelson and Sommer (1996). Phosphorus and potassium were quantified by the procedure of John (1970) through flame photometer-128 (Systronics) after digesting samples in a diacid suspension (HClO_4_: HNO_3_ in the ratio of 4:1). pH and electric conductivity (EC) were determined in double-distilled water blend each with a concentration ratio of 1:10 (w/v) plying digital meter (COM-100) and Eqip-tronics (EQ-614A) respectively. Nitrogen (N) was determined by the Micro-Kjeldhal method as described by Bremner and Mulvancy (1982) using digestion extract (H_2_SO_4_ + K_2_SO_4_: CuSO_4_: SeO_2_ in the ratio of 10:4:1). Phosphorus (P) content was determined by nitro-vanadomolybdate method, potassium (K) by using photometry and micronutrients (Zn and Fe) by atomic-absorption spectrometry (AAS) after digestion of both coprolite samples from GS and GM with HNO_3_:HClO_4_ by the method^113^. Diacid mixture digested samples were analyzed for transition metal elements using an atomic absorption spectrophotometer (Electronic Corporation of India). 

### Statistical analysis

Analysis of variance (ANOVA) was used to compare the results between the control and treatment groups followed by post hoc analysis. A significance level of *P* < 0.05 was used to determine significance in the treatment means using R-software. Regression analysis was carried out using an equation (y $$= b_{2} x^{2} - b_{1} x + a)$$ and to workout maximum responses in different study parameters were determined from the formula $$\left( {x = - b_{1} /2b_{2} } \right)$$. In the experiment, the mean differentiation of chemical parameters of nitrogen dynamics was done using student’s t-test. Parameters such as pH, moisture content (MC), total organic carbon (TOC), total phosphorus (TP), nitrates, total nitrogen (TN), potassium (K), iron (Fe), zinc (Zn), and the carbon and nitrogen ratio (CN ratio) were utilized to determine correlation matrices affecting coprolites’ quality and stability. The findings were plotted and tabulated using principal component analysis.

## Conclusion

The study confirms that GM coprolites contains more nitrogen (NH_4_^+^-N and NO_3_^–^-N) than the GS, which is readily logical when the selective feeding habits of earthworms are considered. At low temperature, simultaneous heterotrophic nitrification and aerobic denitrification (SHNAD) was found to be stable processes and main N transformation mechanism in both substrates. *A. caliginous* promotes microorganism growth, which would otherwise be severely limited due to harsh winter and low ambient temperature. The study highlights the importance of the SHNAD as a pilot scale process showing positive interaction with *A. caliginous* contributing in physico-chemical parameters of its coprolites and thus has substantial potential for N removal from wastes at low temperatures. Interaction between psychrophillic earthworms and microbial genera need to be further investigated to provide insight evidences of co-occurrence pattern of both, which could help to minimize NH_3_ emission by effectively reducing N_2_O.

## Supplementary Information

Supplementary Information.

## Data Availability

The data sets generated are available as supplementary file.
